# Glucose and Loss of Control Eating: Evidence From Naturalistic Assessment After Roux‐en‐Y Gastric Bypass

**DOI:** 10.1002/erv.70102

**Published:** 2026-04-22

**Authors:** Gail A. Kerver, Kristine J. Steffen, Debra L. Safer, Glen Forester, David B. Sarwer, Stephen A. Wonderlich, Scott G. Engel

**Affiliations:** ^1^ Center for Biobehavioral Research Sanford Research Fargo North Dakota USA; ^2^ Department of Psychiatry and Behavioral Science, School of Medicine and Health Sciences University of North Dakota Fargo North Dakota USA; ^3^ Department of Psychology Indiana University Indianapolis Indianapolis Indiana USA; ^4^ Department of Pharmaceutical Sciences North Dakota State University Fargo North Dakota USA; ^5^ Department of Psychiatry and Behavioral Sciences Stanford University School of Medicine Stanford California USA; ^6^ Temple University College of Public Health Philadelphia Pennsylvania USA

**Keywords:** continuous glucose monitoring (CGM), ecological momentary assessment (EMA), loss of control (LOC) eating, metabolic and bariatric surgery (MBS), Roux‐en‐Y gastric bypass (RYGB)

## Abstract

**Objective:**

While loss of control (LOC) eating is associated with poor outcomes following metabolic and bariatric surgery (MBS), the mechanisms driving it are less understood. This study used momentary, naturalistic data to examine glucose as a biological correlate of LOC eating after Roux‐en‐Y gastric bypass (RYGB).

**Method:**

Participants (45 adults one‐year post‐RYGB) completed 10 days of ecological momentary assessment (EMA) while wearing a continuous glucose monitor (CGM). Eating episodes reported in EMA were time‐matched to CGM readings 3 hours before and after eating episodes. Four CGM metrics (mean, variability, time in hyper‐ and hypo‐glycaemia) were used in multilevel models to test whether glucose functioning was a significant antecedent and/or consequence of LOC eating.

**Results:**

Greater mean glucose levels and variability each significantly predicted engagement in an eating episode, while more time spent in hyperglycemia predicted the absence of eating. Significantly higher mean glucose was observed after eating. No glucose metric specifically predicted engagement in LOC eating; however, LOC eating resulted in significantly greater glucose variability.

**Conclusions:**

Glucose level and variability predicted subsequent eating, but not LOC eating. However, LOC eating was associated with subsequent glucose variability, which might help explain how LOC eating is associated with poorer outcomes following RYGB.

## Highlights

- By combining ecological momentary assessment (EMA) with continuous glucose monitors (CGM), this study found that greater mean glucose and glucose variability each significantly predicted engagement in an eating episode after Roux‐en‐Y gastric bypass (RYGB).
- More time spent in hyperglycemia predicted the absence of eating.
- While no glucose metric significantly predicted loss of control (LOC) eating, LOC eating resulted in significantly greater glucose variability.

## 1 Introduction and Aims

Binge eating, a transdiagnostic eating disorder symptom, is now characterised in the 11th revision of the International Classification of Diseases (ICD‐11) primarily by the experience of loss of control over eating regardless of the amount of food consumed (Claudino et al. 2019). Such binge‐eating episodes, otherwise referred to as ‘loss of control (LOC) eating’, have been associated with elevated levels of eating disorder‐related distress and impairment (Goldschmidt 2017; Wolfe et al. 2009). In particular, LOC eating is a concern for persons who undergo metabolic and bariatric surgery (MBS), which is currently the most effective means for substantial weight loss (i.e., average total body weight losses of 25%–30%) and improvement in co‐occurring medical problems (e.g., type 2 diabetes, dyslipidemia, hypertension) among individuals with severe obesity (Klair et al. 2023; Sarma and Palcu 2022; Arterburn et al. 2020; Maciejewski et al. 2016). Approximately 480,000 surgeries were performed worldwide in 2023, with the majority being either the sleeve gastrectomy (SG) or Roux‐en‐Y gastric bypass (RYGB) procedure (International Federation for Surgery for Obesity and Metabolic Disorders 2023). While the benefits of MBS are generally durable, LOC eating has been shown to contribute to less weight loss and weight recurrence over time (Kerver et al. 2025; Conceição et al. 2024; Wimmelmann et al. 2014; Mauro et al. 2019; Kofman et al. 2010). It is estimated that approximately 30%–40% of patients will experience recurrent LOC eating following MBS (White et al. 2010; K. E. Smith et al. 2019). Yet, the aetiology of the development of LOC eating among patients post‐MBS is not well understood. Identifying mechanisms of post‐MBS LOC eating would allow for the development and refinement of postoperative interventions to promote optimal weight outcomes over time (Kober and Boswell 2018; Giel et al. 2022).

Glucose has long been considered a potential biological mechanism for binge eating‐type behaviour. In 1994, Johnson and colleagues observed momentary changes in glucose among women with bulimia nervosa in a controlled laboratory setting, finding that purging produced hypoglycemia (i.e., periods of time wherein glucose levels fall below 70 mg/dL), which then fuelled the binge‐purge cycle (Johnson et al. 1994). Type 2 diabetes, which is seen in a large minority of patients presenting for MBS, has also been linked to elevated rates of binge‐eating disorder (Abbott et al. 2018; Herpertz et al. 1998), further suggesting a relationship between dysregulated glucose and binge eating. Importantly, contemporary research using advanced digital data collection methods has provided evidence of momentary and ecologically‐valid relationships between glucose functioning and disordered eating behaviour (Uotani et al. 2022; Presseller et al. 2020; L. B. Smith et al. 2025). For example, greater disordered eating behaviours measured using ecological momentary assessment (EMA) predicted greater glucose variability measured via continuous glucose monitoring (CGM) among individuals with binge‐spectrum eating disorders (Presseller et al. 2022). Machine learning algorithms using CGM data have also been trained to accurately identify the occurrence of binge eating and vomiting (Presseller et al. 2024).

RYGB is considered the primary MBS procedure for use in the treatment of type 2 diabetes among individuals with severe obesity (Meijer et al. 2011). Indeed, individuals who undergo RYGB typically experience significant improvement in glycaemic control, which is believed to occur via weight‐dependent and independent mechanisms (Sandoval and Patti 2023; Sjöholm et al. 2016; Mingrone et al. 2021). However, RYGB MBS also increases the risk for recurrent hypoglycemia (Sandoval and Patti 2023; Salehi et al. 2018), with estimates drawn from CGM data suggesting that over 50% of patients will experience hypoglycemia at some point postoperatively (Lupoli et al. 2022; H. Zheng et al. 2025). These experiences may prompt patients to eat in an unplanned or ‘maladaptive’ manner (Roslin et al. 2011, 2013; Maleckas et al. 2016), thus suggesting that momentary dysregulation of glucose could be a particularly salient mechanism of post‐RYGB LOC eating. However, to our knowledge, the link between momentary glucose functioning and post‐RYGB LOC eating in the natural environment has not yet been investigated.

This study utilised naturalistic assessment methods to explore the prospective relationships between multiple aspects of glucose functioning and LOC eating following RYGB MBS. The study also compared the prospective relationships between glucose functioning and non‐LOC eating episodes as well as time‐matched periods with no eating. We hypothesised that there would be a unique effect of glucose on LOC eating compared to non‐LOC eating and periods of no eating, and that the glucose response following episodes of LOC eating would be markedly distinct from the glucose response following non‐LOC eating and periods of no eating.

## 2 Methods

### 2.1 Participants and Procedures

Adults who underwent the RYGB surgical procedure approximately 1 year prior to the study (i.e., between 11 and 15 months post‐surgery) were recruited from the affiliated hospital's metabolic and bariatric surgery clinic. Participants were ineligible for the study if they had a current diagnosis of type I or II diabetes mellitus, were taking medications for the control of blood glucose, or had received treatment for hypoglycemia in the past 3 months. Participants were also excluded if they were actively involved in weight loss treatment beyond RYGB (e.g., supervised dietary intervention, weight loss medication), received a revision to the RYGB procedure, or were pregnant or lactating.

Following informed consent and confirmation of eligibility in the laboratory, participants completed a series of baseline self‐report questionnaires and had a CGM device (see below for additional details) placed on their lower abdomen by a registered nurse research specialist. Once the CGM device was paired with the study computer tablet, participants were instructed on how to complete the EMA protocol over the next 10 days.

During the 10‐day EMA protocol, a signal indicating that the participant needed to complete a questionnaire was delivered six times each day between the hours of 8 a.m. and 10 p.m. If participants did not respond to a signal within 30 min, then the signal was considered as missing data. Participants were instructed not to complete signals at times in which it may be unsafe to do so (e.g., while driving), and were shown how to activate the ‘do not disturb’ function on the EMA application, as needed. All research procedures received institutional review board approval.

### 2.2 Materials

#### 2.2.1 Computer Tablet

To complete the EMA protocol, participants were provided with a computer tablet. The Mobile Assessment Tool (MAT) (Dvorak et al. 2018) software application was downloaded onto the computer tablet and used to run the EMA protocol.

#### 2.2.2 Continuous Glucose Monitor

Participants wore a Dexcom G6 Pro continuous glucose monitor (CGM) system (Dexcom Inc.; San Diego, CA, USA) on their abdomen. Glucose readings (in mg/dL) were obtained from interstitial fluid every 5 min for the duration of the 10‐day wear period. The CGM was connected to a data management software application (Integrated Medical Development LLC; Princeton Junction, NJ, USA) housed on the computer tablet via a Bluetooth wireless connection. To minimise reactivity to the study protocol, participants were blinded to glucose readings (i.e., could not see glucose readings on the computer tablet) for the duration of the CGM wear period.

### 2.3 Measures

#### 2.3.1 Eating Behaviour

Within each EMA signal, participants were asked to indicate whether they ate since they were last signalled. If the participant endorsed an eating episode, they were asked to indicate (in 30‐min time increments) how long ago the episode occurred. Subsequently, the extent to which the participant experienced LOC over their eating was assessed using three items previously developed for the momentary, naturalistic assessment of binge eating (see Table 1) (Goldschmidt et al. 2014; Peterson et al. 2020). For analyses, a LOC eating episode was defined by the occurrence of an eating episode accompanied by a score ≥ 3 on at least one of the three LOC items. Scores on the overeating item were included as a covariate in prospective analyses.

**TABLE 1:** Eating and LOC eating episode questions in EMA.

Question	Response option
Eating episode	
1. Have you eaten or drank anything since the last assessment?	Yes or no
(If ‘Yes’, branch to the following questions)	
2. When did you start eating?	Multiple choice: 0–30 min ago 31–59 min ago 1–1.5 h ago 1.5–2 h ago Over 2 h ago
LOC eating	
3. While you were eating, to what extend did you feel a sense of loss of control?	Likert scale: 1 = Not at all 2 = A little 3 = Moderately 4 = Quite a bit 5 = Extremely
4. While you were eating, to what extent did you feel that you could not stop eating once you started?	Likert scale: 1 = Not at all 2 = A little 3 = Moderately 4 = Quite a bit 5 = Extremely
5. While you were eating, to what extent did you feel driven or compelled to eat?	Likert scale: 1 = Not at all 2 = A little 3 = Moderately 4 = Quite a bit 5 = Extremely
Overeating	
6. While you were eating, to what extent do you feel that you overate?	Likert scale: 1 = Not at all 2 = A little 3 = Moderately 4 = Quite a bit 5 = Extremely

Abbreviation: LOC = loss of control.

#### 2.3.2 Glucose Monitoring

To characterise patterns of glucose, four objective measurements (herein referred to as ‘metrics’) were calculated based on recommendations for the use of CGM data in clinical research trials (Battelino et al. 2023). Glucose readings (in mg/dL) within a three‐hour window of time were used for calculations (see ‘Data Preparation and Analytic Plan’ section for further detail). Average (‘mean’) glucose was calculated by summing the glucose concentration values within the three‐hour window and then dividing by the number of readings in that time period. ‘Variability’ of glucose was calculated as the standard deviation (SD) of glucose values within the three‐hour window. Time in range estimates were calculated for hypoglycemia (‘% hypoglycemia’; i.e., the percentage of the three‐hour window of time spent with glucose < 70 mg/dL) and hyperglycemia (‘% hyperglycemia’; i.e., the percentage of the three‐hour window of time spent with glucose > 180 mg/dL).

### 2.4 Data Preparation

The date and time of data were matched between the EMA protocol and CGM device. Given that the timing of an eating episode could have occurred within a 30‐min window of time, the earliest and latest possible timestamp for the episode was calculated by subtracting the selected time window from the timestamp of the EMA signal. For example, if a signal was completed at 3:00 p.m., and the participant indicated that an eating episode occurred 31–59 min ago, then the eating episode was estimated to have occurred between 2:01 p.m.–2:29 p.m. In order to match glucose data with an eating episode, the midpoint time of the eating episode was identified (e.g., 2:15 p.m. in the aforementioned example), and all glucose data in the 3 hours prior to the midpoint (e.g., 11:15 a.m.–2:15 p.m.) were used for the ‘pre‐eating’ analyses, and the 3 hours following the midpoint (e.g., 2:15 p.m.–5:15 p.m.) were used for the ‘post‐eating’ analyses.

All valid glucose readings within the three‐hours pre‐ or post‐eating were utilised in each glucose metric calculation. For comparisons between eating and no‐eating episodes, glucose values in the 3 hours immediately prior to the EMA signal were used for no‐eating episodes. If additional eating episodes occurred within the designated three‐hour timeframe for analysis, then those episodes were entered as a binary covariate. To isolate effects at the momentary level, and control for any potential between‐person confounding factors, each glucose metric was within‐person centred. Thus, each glucose metric used in analyses represents how an individual glucose event (e.g., pre‐eating mean glucose) compared to the individual's own baseline (e.g., their three‐hour mean glucose across all 10‐day of the protocol). Consistent with the manufacturer instructions, CGM readings ≤ 40 mg/dL or ≥ 400 mg/dL were considered erroneous and recoded as missing data prior to analysis.

### 2.5 Data Analysis

Multilevel modelling (Bauer 2003) was used to test the prospective relationships between patterns of glucose and eating episodes, as this approach allows for momentary data to be nested within‐person. Four sets of analyses were conducted. First, to test if a prior pattern of glucose predicted the likelihood of subsequent eating (i.e., ‘pre‐eating’), we fit a model that included the four glucose metrics (i.e., mean, variability, % hyperglycemia, % hypoglycemia) as predictors and subsequent eating (vs. not eating) as a dichotomous outcome. The time of day and whether any additional eating episodes took place during that timeframe were entered as covariates. Second, we tested the relationship between prior eating and subsequent patterns of glucose (i.e., ‘post‐eating’) using a separate model for each of the four glucose metrics as outcome variables. In each model, prior eating (vs. no eating) was entered as the predictor, and time of day and whether any additional eating episodes took place during the specified timeframe were entered as covariates. Third, we repeated the ‘pre‐eating’ model, but utilised the LOC eating (vs. non‐LOC eating) variable as the outcome of interest. This ‘pre‐LOC eating’ model controlled for the presence of overeating during the eating episode, time of day, and whether any additional eating episodes took place during the specified timeframe. Finally, we repeated the ‘post‐eating’ model but used the LOC eating (vs. non‐LOC eating) variable as the predictor of the four subsequent glucose metrics. Each ‘post‐LOC eating’ model controlled for the presence of overeating during the eating episode, time of day, and whether any additional eating episodes took place during the specified timeframe.

**FIGURE 1:**
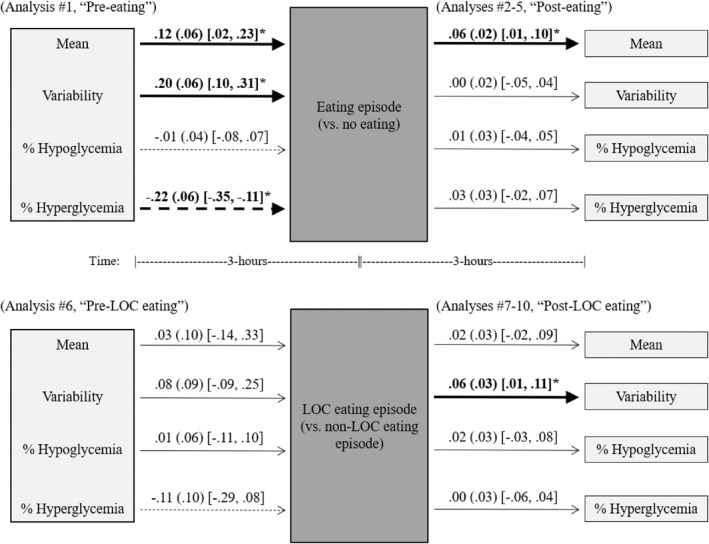
The presented model depicts the results of the four analyses proposed in the current study. The statistics provided include the model estimate, (Posterior standard deviation), and [Bayesian credibility interval]. Solid arrows indicate positive associations, and dashed arrows indicate negative associations. Bolded arrows and statistics denoted with an asterisk indicate statistically significant effects. Of note, the post‐eating and post‐LOC eating variability results depicted in the figure use the SD calculation. Sensitivity analyses using the coefficient of variation ‘CoV’ produced very similar effects for post‐eating and post‐LOC eating: −0.01 (0.02) [−0.06, 0.04] and 0.06 (0.03) [0.01, 0.12]*, respectively. LOC = loss of control.

Given the lack‐of‐consensus in the literature regarding the most appropriate measure of glucose variability (Suh and Kim 2015), we ran additional sensitivity analyses using another recommended metric—the coefficient of variation (‘CoV’ = SD/M × 100) (Battelino et al. 2023; DeVries 2013). This measure was utilised in post‐eating and post‐LOC eating models only, as including it in the single pre‐eating and pre‐LOC eating models would result in problematic multicollinearity. The pattern of results remained consistent across the post‐eating and post‐LOC eating models predicting the CoV (see Figure 1). Thus, we retained the standard deviation (SD) calculation for variability to maintain consistency across pre‐ and post‐analyses.

All analyses were conducted using Mplus 8.11 (T. Muthén and Muthén 2017) with Bayesian estimation (B. Muthén and Asparouhov 2012). Effects were categorised as statistically significant when the 95% Bayesian credibility interval (BCI) did not include zero. Parameter estimates are reported with their respective posterior SDs, which are analogous to the standard errors provided when using frequentist methods of estimation (McNeish and Hamaker 2020).

## 3 Results

### 3.1 Demographic and Descriptive Variables

The sample was comprised of 45 adults (38 females, 84.4%) with an average age of 43.44 years (SD = 8.51). The majority of the sample identified as White (*n* = 43, 95.6%), with the remaining two participants identifying as either Black (2.2%) or American Indian/Alaska Native (2.2%). The average body mass index (BMI) was 29.45 kg/m^2^ (SD = 4.75).

Participants reported a total of 1652 eating episodes during the EMA protocol (10‐day person‐level mean = 36.7 episodes, SD = 10.1), including 190 LOC eating episodes (10‐day person‐level mean = 4.2 episodes, SD = 7.5). Average three‐hour glucose levels per person was 122.0 mg/dL (SD = 12.4), mean glucose variability (i.e., standard deviation) was 21.8 mg/dL (SD = 6.3), mean % time spent in hypoglycemia was 1.0% (SD = 1.6), and mean % time spent in hyperglycemia was 6.4% (SD = 5.6). Overall adherence to the EMA protocol, calculated as the percentage of signals completed relative to the total number of signals delivered, was 83%.

### 3.2 Prospective Relationships Between Glucose and Eating Episodes

#### 3.2.1 Eating Versus No Eating

Analysis #1 (‘Pre‐eating’) tested whether the within‐person centred glucose metrics calculated from CGM data during the 3 hours preceding an eating episode predicted the likelihood of eating versus not eating (see Figure 1). Results demonstrated that three glucose metrics—mean, variability, and % hyperglycemia—were all uniquely predictive of subsequent eating. Specifically, greater mean glucose and greater glucose variability (compared to the individual's own baseline levels of mean glucose and glucose variability, respectively) in the 3 hours prior to an eating episode were associated with an increased likelihood of eating (*B* = 0.12 and *B* = 0.20, respectively). Alternatively, greater time spent in *hyper*glycemia in the 3 hours prior to eating (compared to the individual's baseline level of time spent in hyperglycemia) was associated with a decreased likelihood of eating (*B* = −0.22). The effect of time spent in *hypo*glycemia was not statistically significant. Analyses #2–5 (‘Post‐eating’) then tested whether an eating episode predicted patterns of glucose responding in the 3 hours following the eating episode. Results showed that eating significantly predicted greater mean glucose (compared to the individual's baseline level of mean glucose; *B* = 0.06). The impact of eating on glucose variability, % hypoglycemia, and % hyperglycemia was not significant.

#### 3.2.2 LOC Versus Non‐LOC Eating

Analysis #6 (‘Pre‐LOC eating’) tested whether within‐person centred 3‐h glucose metrics predicted the subsequent occurrence of LOC eating versus non‐LOC eating (see Figure 1). Results failed to detect a significant relationship between the glucose metrics and LOC eating. Analyses #7–10 (‘Post‐LOC Eating’) then tested whether a LOC eating episode predicted 3‐h patterns in glucose responding immediately following the LOC eating episode. Results demonstrated that LOC eating was associated with greater subsequent glucose variability (compared to the individual's baseline levels of glucose variability; *B* = 0.06). Results for glucose mean, time spent in hypoglycemia, and time spent in hyperglycemia were not significant.

### 3.3 Post‐Hoc Analysis

Given that LOC eating predicted greater glucose variability in the 3 hours following the episode compared to non‐LOC eating episodes, we questioned whether this variability could possibly represent dumping syndrome wherein glucose levels quickly spike following food consumption but then rapidly fall to hypoglycemic levels (Sandoval and Patti 2023) (see Discussion for additional information). Further, glucose variability emerged as a significant predictor of eating versus no eating, which led to the formation of a secondary hypothesis that post‐LOC eating glucose variability could prompt a subsequent episode of eating during the same three‐hour window of time. To explore this, a post‐hoc analysis was conducted utilising the same multilevel modelling approach as analyses #7–10 except that, instead of controlling for subsequent episodes of eating, the additional episodes of eating were entered as the outcome in the model. While subsequent eating episodes were slightly more common following a LOC eating episode (41%, SD = 0.30%) compared to a non‐LOC eating episode (38%, SD = 0.10%), the results of the multilevel model suggest that the difference was not statistically significant: 0.025 (0.13) [−0.202, 0.293].

### 3.4 Discussion

This study sought to add insight into a possible biological mechanism of post‐RYGB MBS LOC eating by employing a unique combination of ambulatory assessment methodology to examine the bidirectional relationship between glucose and LOC eating. Results showed no glucose metric significantly predicted LOC eating; however, LOC eating resulted in significantly greater glucose variability. These results, paired with several significant glucose effects for eating versus no eating, have possible implications towards identification and treatment of post‐RYGB MBS LOC eating.

Low blood glucose levels (i.e., hypoglycemia) often trigger increases in hunger and craving of carbohydrates (Anderson et al. 2002; Iqbal and Heller 2016). In people who have undergone RYGB, this effect of hypoglycemia may help to explain why patients go on to engage in unplanned eating that is accompanied by feelings of loss of control after surgery. However, in the current study, hypoglycemia did not predict eating of any kind (i.e., neither LOC eating or non‐LOC eating); no glucose metric was a significant predictor of LOC eating over non‐LOC eating. Instead, specific patterns of glucose were more likely to predict whether someone would engage in any type of eating (i.e., regardless of loss of control or other psychological indicators of disordered eating).

More time spent in hyperglycemia resulted in a decreased likelihood that someone would engage in eating. This finding is consistent with literature suggesting that appetite and the drive for eating is dampened when glucose levels are high to allow the opportunity for glucose to decrease and return to homoeostatic levels (Louis‐Sylvestre 1999; Mayer 1953). Alternatively, higher mean glucose levels and greater glucose variability in our participants were significantly associated with an increased likelihood of eating. While this finding initially appears counterintuitive, it may highlight that RYGB results in altered and unique relationships between glucose dynamics and eating behaviour, similar to that observed among patients with type 2 diabetes (Knudsen et al. 2014), that warrant further and more nuanced investigation.

Typically, glucose levels rise after food consumption, and that higher glycaemic index foods (such as pasta, white rice, and starchy vegetables) produce a more exaggerated glucose response (Louis‐Sylvestre 1999; Brand‐Miller et al. 2003; Fabricatore et al. 2011). Thus, it was unsurprising to find in our data that eating resulted in higher mean glucose levels compared to no eating. However, a different consequent effect also emerged ‐ engagement in a LOC eating episode predicted significantly greater glucose variability over the 3 hours following the episode compared to episodes of eating without significant levels of LOC. This suggests that, instead of simply raising glucose levels, LOC eating resulted in significant fluctuations in glucose levels over the 3 hours following the eating episode. This finding is consistent with the results of Presseller and colleagues (Presseller et al. 2022) who found an association between binge eating behaviours and glucose variability. The current study extends upon this finding to elucidate the association of LOC eating and glucose variability within a post‐RYGB MBS population.

We question whether the pattern of post‐LOC eating glucose variability could possibly mirror the glucose response associated with post‐MBS dumping syndrome (Tack et al. 2025; Patti and Goldfine 2016). While the exact pathophysiology of dumping syndrome has yet to be confirmed, one theory is that sharp rises in glucose following the consumption of high glycaemic‐index foods leads to a rapid decrease in glucose (Patti and Goldfine 2014; Goldfine et al. 2007). This ‘rollercoaster’ effect, as measured via CGM, has been associated with the occurrence of dumping syndrome (Patti and Goldfine 2016; Ri et al. 2021). Indeed, a link between dumping syndrome and disordered eating behaviours, including LOC eating, has been previously suggested (Müller et al. 2024; Kalarchian et al. 2017). While additional information (e.g., evidence of Whipple's triad) would be needed to confirm the occurrence of dumping syndrome (Patti and Goldfine 2016; Emous et al. 2015), our data could potentially demonstrate a direct link between disordered eating behaviour and the glucose variability associated with dumping syndrome following RYGB.

Given that LOC eating predicted greater glucose variability in the 3 hours following the episode, paired with the finding that greater glucose variability predicted eating over no eating, we began to wonder whether our data suggested a cyclical effect wherein LOC eating would prompt subsequent eating episodes as a means of regulating glucose variability. However, the results of our post‐hoc analysis failed to support this secondary hypothesis, suggesting that LOC eating was not more likely to be followed by a subsequent eating episode than were non‐LOC eating episodes. Thus, the glucose variability resulting from LOC eating episodes may highlight a specific risk factor for poor outcomes following RYGB MBS. Indeed, a recent review of the use of CGM following MBS highlighted that glycaemic variability following MBS can lead to oxidative stress, a known risk factor for cardiovascular disease (Bally et al. 2023). The relationship between glycaemic variability and oxidative stress underscores the importance of monitoring eating patterns in post‐MBS patients, as understanding these patterns can help healthcare providers develop targeted interventions to mitigate the risks associated with disordered eating and its physiological consequences.

Currently, there is a lack of agreement in the MBS literature regarding classification and measurement of post‐MBS disordered eating behaviours, particularly binge eating versus LOC eating (Kerver et al. 2025; Ivezaj et al. 2021). Postoperatively, the reduced stomach size prevents patients from eating the large amount of food required for the diagnosis of BED. However, patients often report feelings of loss of control in the absence of eating large amounts of food. LOC eating appears to be the more relevant marker of postoperative eating pathology (Goldschmidt et al. 2016; Ivezaj et al. 2018). However, one difficulty with properly identifying LOC eating is that patients often experience shame and guilt regarding these eating episodes, making it more difficult for patients to accurately report their occurrence. Identification of empirically supported objective indicators of LOC eating could thereby become critical in the future study of disordered eating following MBS. To this end, this study is the first to find evidence for an objective and unique signature of LOC episodes in post‐RYGB patients. While further investigation among larger samples of patients is needed to confirm this objective signature, such an ability to passively receive information about an LOC episode from CGM data could help increase awareness of this risk factor for poor postsurgical outcomes and provide a basis for early intervention without reliance on self‐report.

Strengths of this study include the combination of ambulatory assessment methods—CGM and EMA. While there is growing use of these methods both in the eating disorders and MBS literature, no known studies have employed both methods to study post‐RYGB MBS LOC eating. The results from this study, in addition to similar studies in the eating disorders field (Uotani et al. 2022; Presseller et al. 2022, 2024), help underscore the potential for these technologies to aid research and clinical work. For example, future studies may seek to explore whether EMA and CGM methods could be incorporated into just‐in‐time adaptive interventions (JITAIs) (Nahum‐Shani et al. 2018) aimed at preventing and intervening upon disordered eating behaviours in the moment (Presseller et al. 2020, 2024, 2023). Another strength of this study is that participants were blinded to their glucose readings. This blinding helps minimise the potential for reactivity bias in report of LOC eating, particularly among patients who may have had previous experience with glucometers to regulate eating behaviour (e.g., those with a presurgical diagnosis of diabetes). The final strength of this study is the use of analytic methods that isolate changes in glucose dynamics within individuals over time. Many factors that differ between individuals—such as haemoglobin A1c or hormones like ghrelin, leptin, and GLP‐1—can influence glucose dynamics. Thus, analysis approaches that aggregate across (rather than within) individuals are likely to confound these between differences with the within‐person relationships of interest. By assessing each person's glucose dynamics at a given moment relative to their own typical glucose levels, we were able to more accurately capture how shifts in an individual's glucose dynamics relate to LOC eating.

Despite these methodological strengths, there are also several limitations. First, as mentioned earlier, the exact timing of eating episodes, including LOC eating, was not captured during the EMA protocol, which precludes more detailed analyses of glucose functioning. Second, haemoglobin A1c values were not obtained from participants. This could have been an informative covariate in the analyses; although, the within‐person analyses conducted may help mitigate some of these concerns by controlling for individual baseline differences. Third, the CGM device used in this study may yield higher readings for hypoglycemia compared to other methods (Y. Zheng et al. 2025). However, this sensitivity also meant that episodes of hypoglycemia, when present, were unlikely to be missed. Fourth, information was not obtained about other factors known to influence glucose variability, such the macronutrient content of the food consumed during LOC eating and non‐LOC eating episodes. Further, some LOC eating episodes may not have been reported because the EMA protocol ended each day at 10 p.m.

The number of individuals who undergo MBS annually, and the sizeable minority who experience these issues postoperatively, justify further evaluation of these relationships. For example, studies examining the impact of glucose functioning on disordered eating following MBS should seek to record the macronutrient intake (e.g., carbohydrate density) associated with LOC eating. Similarly, future EMA work in this area could seek to utilise event‐contingent responding, thus allowing participants to self‐initiate an EMA survey when they engaged in LOC eating. This would provide a more precise timestamp for the LOC eating episode, which would then allow for advanced analytic techniques to be conducted on glucose data (e.g., calculating area under the curve to better confirm the occurrence of dumping syndrome). Future research may also aim to gather information on appetitive hormones (e.g., leptin, ghrelin, PYY, GLP‐1) to gain insight into the relationships between these hormones and glucose functioning as it relates to eating behaviour following MBS.

In summary, although our results failed to find a significant pattern of glucose functioning on LOC eating following MBS, the consequent effect of greater glucose variability emerging following LOC eating contributes novel information to our understanding of the mechanisms of LOC eating following MBS. The findings also represent a meaningful step forward in better elucidating why and how LOC eating promotes poorer weight outcomes following MBS.

## Funding

Research reported in this publication was supported by the National Institute of General Medical Sciences of the National Institutes of Health under Award Number P20GM134969. The content is solely the responsibility of the authors and does not necessarily represent the official views of the National Institutes of Health.

## Ethics Statement

This research received approval from the Sanford Health Institutional Review Board (approval #STUDY00002278).

## Conflicts of Interest

The authors declare no conflicts of interest.

## Data Availability

The datasets generated during and/or analyzed during the current study are available from the corresponding author on reasonable request.
